# If We Had State-Aided Hospitals

**Published:** 1903-03-21

**Authors:** 


### HOSPITAL ADMINISTRATION.

### CONSTRUCTION AND ECONOMICS.

### IF WE HAD STATE-AIDED HOSPITALS.

To the enthusiastic reformers who would like to put everything, including our hospitals, under the State, we would recommend a perusal of the report of the New York County Visiting Committee, presented to the State Charities Aid Association. We have in this country no such association and no such committee, because we have so many voluntary charities, and where support is voluntary there is always more or less control by subscribers. Where this is lacking, the charitable public—by which we mean those who interest themselves in their poorer brethren rather than those who only give money—have cause to be thankful that there is such a committee. It has, as its report declares, "no official connection with the institutions; it knows no consideration of party, race, or creed; and visits for the purpose of finding out how the dependent of the city are cared for, and of assisting, in whatever ways may be possible to private citizens, in improving the position of such dependents. Its membership is independent of official appointment; its expenses are met by voluntary contributions of its members and friends, and its sole legal power is that of free access to all parts of the institutions upon orders issued by the Justices of the Supreme Court upon application by the President of the Association." It is well that such a committee should exist, and it has done much good in drawing attention to defects in the institutions which it visits, but various details show how much such supervision is needed.

Thus in the Bellevue Hospital, which is an ordinary general hospital, although, being a State institution, it has wards for sick prisoners, and also "for the examination of the alleged insane"—a rather curious addition to its general purpose—we are told that "the demand for better quarters for the employees of the institution is urgent, as almost the worst buildings on the grounds, and much the most undesirable portions of the main building are used by them as dormitories. The women are herded in open dormitories in the basement of the main building, the attic of the old Alcoholic Pavilion, and the rooms of the old Cornell Medical College building, where privacy is impossible, where no wardrobes or lockers are provided for clothing and where vermin abound. The dormitories for male helpers are in other parts of the basement of the main building, in the old Alcoholic Pavilion and on the second floor of the building used for patients' clothing, which was formerly the ambulance stable. The dormitories in the basement of the main building are absolutely dark, even in the middle of the day; are insanitary and overrun with vermin. There are no sitting-rooms, either for the men or the women employees." No wonder that in another part of the report we are told that "it is very difficult to persuade respectable helpers to occupy the quarters now furnished." Certainly the Bellevue Hospital has been in a bad way of late, for we read that the trustees of the hospital "have removed the registrar because of intemperance, and the steward on the ground of incompetence." It is to be hoped that under new management the administration of the hospital will be more satisfactory. Male nurses are still employed in the male wards of the hospital, but they are now being put under the charge of a female head nurse. As orderlies they may be useful, but it is evident that the class from which they are drawn is not a very high one, for we are told that even employees of the institution were among those who availed themselves of the Alcoholic Pavilion as a place where they could "sober off." The boast of the committee that a smaller hospital, "Gouverneur," with a hundred beds is "the peer of many private institutions" is a touching confession of the admitted difference in comfort and efficiency which obtains in the latter.

While, however, we do not think that the report of the State-aided Charities of New York encourages a similar experiment on this side of the Atlantic, we admit that the Department of Public Charities there has taken a step which deserves imitation. They have opened a Tuberculosis Infirmary. In the existing hospitals under the Department, there were, on January 1st, 1902, 318 consumptive patients, 158 of whom were distributed through wards occupied by other patients, while the remainder were in separate wards, but under the same roof as the general patients. If tuberculosis is admitted to be an infectious disease, there is no doubt that it should be put under the same regulations as other infectious diseases, and patients suffering from any form of it should be segregated. The duty of the State to take every means to prevent infection in the interests of public health, is obvious, although it is to be regretted that in the case of phthisis there is every probability of a patient having infected his family before he enters hospital at all. But that is no reason for allowing him to infect his fellow-patients.

Another improvement made within the year will commend itself to those who realise that the respectable poor, when they have to apply for State aid, suffer much from the rough manner of those by whom they are examined. "It is the purpose of the superintendent, as the representative of the department (of the outdoor poor), to consider each application for relief carefully, impartially, individually, and in such a way as not to wound the sensibilities of the unfortunate. Accordingly, a woman examiner has been assigned to hear privately all statements made by women in abandonment and bastardy cases, which hitherto have been made publicly to the superintendent." It is possible that many of these cases deserve more sympathy than similar ones in this country. The constant emigration to America, which places on its shores annually thousands who know nothing of its laws, facilitates the opportunities of unscrupulous men to ruin ignorant girls. The latter may believe themselves legally married according to American law, when only some mockery of a ceremony has taken place, and such cases deserve sympathy and help rather than condemnation.

That the foreign element is a cause of special difficulty in the administration of poor relief in America may be inferred from the fact that the committee recommend that an interpreter who can speak several languages should be added to the office staff, as "applications are frequently made by persons unable to speak English, and it is with considerable difficulty that they make their wants known. This sometimes results in mistakes or causes increased labour which might be avoided."

Let us hope that it is only in America that it is necessary that an attempt should be made to break up an "undertakers' trust." This trust often subjects the relatives of patients dying in any of the city hospitals to embarrassment and extortion, and as it appears, cases in which mistakes have been made in the delivery of bodies are by no means unknown.

